# Hemivertebra resection after age three produces the similar results but with less complications compared to earlier surgery: a minimum of 5-year follow-up

**DOI:** 10.1186/s13018-023-04096-7

**Published:** 2023-09-02

**Authors:** Yu Wang, Xu Sun, Wenhan Li, Benlong Shi, Zhen Liu, Bin Wang, Yong Qiu, Zezhang Zhu

**Affiliations:** 1 Division of Spine Surgery, Department of Orthopedic Surgery, Nanjing Drum Tower Hospital, Affiliated Hospital of Medical School, Nanjing University, Zhongshan Road 321, Nanjing, 210008 China; 2 Division of Spine Surgery, Department of Orthopedic Surgery, Nanjing Drum Tower Hospital, Clinical College of Nanjing University of Chinese Medicine, Zhongshan Road 321, Nanjing, 210008 China

**Keywords:** Congenital scoliosis, Hemivertebra, Resection, Complication

## Abstract

**Background:**

The optimal timing for hemivertebra resection remains controversial. Early intervention before 3 years of age seems being able to get better correction with less fusion segments. However, it was also reported that early surgery may be associated with more complications. The purpose of this study is to investigate correction outcomes and complications of delayed hemivertebra resection (between 3 and 5 years of age), in comparison with earlier surgery (before 3 years of age).

**Methods:**

Patients who had undergone thoracolumbar hemivertebra resection at a single level before 5 years of age and had more than 5 years of follow-up were reviewed. Twenty-four patients had hemivertebra resection surgery below 3 years of age (early surgery, Group E), and 33 patients received surgery between 3 and 5 years of age (delayed surgery, Group D). Radiographs from preoperative, immediately postoperative, and the latest follow-up visits were reviewed to investigate the correction outcomes. Complications were recorded and compared between these two groups.

**Results:**

The patients of Group E had shorter operation time and less blood loss than those of Group D (*P* = 0.003 and *P* = 0.006). Notably, the fusion segments were 2.3 ± 0.7 and 3.1 ± 1.2 in group E and group D (*P* = 0.005), respectively, indicating group E averagely saved 0.8 motion segments. At the time of surgery, group E had smaller main curve magnitude either in the coronal or in the sagittal plane than group D and experienced similar correction rates of scoliosis (83.3 ± 21.6% vs. 81.2 ± 20.1%, *P* = 0.707) and kyphosis (65.1 ± 23.8% vs. 71.7 ± 24.9%, *P* = 0.319). However, group E had relatively higher complication rates than group D and relatively greater correction loss in either coronal or sagittal plane during follow-up.

**Conclusions:**

Hemivertebra resection resulted in similar correction results in both age groups. However, the rate of complications was lower for Group D than Group E. Thus, for non-kyphotic hemivertebra, surgery may be delayed till 3 to 5 years of age.

## Introduction

Arose as a result of failure of vertebral formation, hemivertebra is the main cause of congenital scoliosis (CS) [1–3]. Since the presence of growth plates cranially and/or caudally, hemivertebra has persistent growth potential similar to a normal vertebra [4]. In this way, hemivertebra could produce awful effect on spinal growth which could lead to asymmetrical longitudinal growth of spine [2]. As a result, scoliosis progression and spinal imbalance are usually expected in CS patients with a non-incarcerated hemivertebra [2]. Concerning the poor prognosis of scoliosis secondary to hemivertebra, surgical intervention is frequently needed to prevent primary curve deterioration and secondary curve development [5–7].

First introduced by Royle, hemivertebra resection has been accepted as the optimal surgical treatment for CS patients with hemivertebra because of the radical removal of deformity driver [8, 9]. Excision of hemivertebra is frequently advocated to be performed as early as possible, with the goals of avoiding deterioration of the rigid deformity and saving compensatory motion segments [5, 7, 10]. It remains controversial how early a CS patient with a thoracolumbar hemivertebra require a surgical intervention. Some surgeons advocated an early surgery even if younger than 3 years old, while others recommended a surgery to be done beyond 3 years. Ruf et al. [5] reported excellent correction outcomes with short fusion segments in 28 CS patients who underwent hemivertebra excision at a young age from 15 months to 6 years and 11 months. In the study from Lazar et al. [11], a total of 11 CS patients were treated by hemivertebra resection before 3 years old and satisfied correction results were achieved with no complications. Thus, they concluded that hemivertebra resection should be performed before 3 years old. Furthermore, it has also been proven that transpedicular screw instrumentation in patients at an early age did not hamper the development of vertebral bodies and spinal canal during the subsequent growth period [5, 12–14]. However, despite the good results in aforementioned studies, young age is always a great challenge for patients to receive hemivertebra resection [15]. Low bone density and small pedicles in young patients might increase the risk of implant-related complications [16]. Additionally, low body weight was reported to be associated with high risk of general anesthesia related complications [17]. Considering the high risk of correction surgery associated with young age, it is also recommended by some spine surgeons that hemivertebra excision should be delayed until the child reached 3 years of age.

Despite the consensus on early surgical intervention for CS patients with a hemivertebra, till now, the optimal timing for hemivertebra resection remains a debate. Thus, this study was conducted to analyze correction outcomes and complications of delayed hemivertebra resection (3–5 years of age) by comparing with earlier surgery (below 3 years of age) and to determine the appropriate timing for hemivertebra resection.

## Materials and methods

### Patients

This study was approved by the ethics committee of our hospital. Young CS patients who were treated by posterior-only thoracolumbar hemivertebra resection and pedicle screw instrumentation from January 2009 to December 2017 were retrospectively reviewed from our scoliosis database. The inclusion criteria were as followed: (1) age ≤ 5 years old; (2) with a minimum of 5-year follow-up; and (3) with complete radiographs. Patients who met the following criteria were excluded: (1) with multiple hemivertebra; (2) having a spinal surgery history; and (3) discrepancy of lower extremities. The demographic and clinical data were recorded according to medical records and operative reports. Preoperative health status was evaluated based on the American Society of Anesthesiologists (ASA) Physical Status Classification System [18]. Based on the age at the time of surgery, patients were subdivided into two groups: Group E (earlier surgery, < 3 years old) and Group D (delayed surgery, between 3 and 5 years old).

### Surgical technique

All of the surgeries were carried out by a surgical team who were experienced with posterior-only hemivertebra resection procedure. The surgical strategy for each patient was designed based on age, curve magnitude, segmental kyphosis, and coronal and sagittal balance. Basically, the fusion level for patients with an isolated hemivertebra was ideally designed from hemivertebra − 1 to hemivertebra + 1 [19]. Exceptionally, in cases with marked kyphotic deformity or thin pedicles, fusion level was extended to hemivertebra ± 2, with the goals to provide strong strength to close osteotomy gap or avoid implant failure.

After general anesthesia, patients were placed in the prone position. A middle incision was made. According to the surgical strategy designed before surgery, the vertebrae which needed to be fused were exposed by subperiosteal dissection. After determination of the position of hemivertebra by intra-operative fluoroscopy, pedicle screws were inserted into the adjacent normal vertebrae using free-hand technique. Then, a pre-contoured rod was temporarily placed on the concave side. The posterior elements of the hemivertebra, including the spinous process, the lamina, the transverse process, and the facet joints, were carefully removed. In case of a thoracic hemivertebra, the rib head was also excised. Subsequently, the body of the hemivertebra was excised completely. The disks above and below the hemivertebra, as well as the contralateral disk, were also completely removed. Intervertebral bone grafting was performed using cancellous bone from the resected hemivertebra. After connection of the other pre-contoured rod on the convex side, compression manipulation was gradually applied on the convex side until the osteotomy gap was closed. The residual cancellous bone was used for posterolateral bone grafting. During surgery, the dural sac and the nerve roots were carefully protected. All of the surgeries were carried out under the neuromonitoring of motor evoked potential (MEP) and sensory evoked potential (SEP). Wearing a plastic brace at least 3 months was prescribed for each of the patients.

### Radiographic measurements

Standing erect posteroanterior X-ray films of the whole spine were obtained before surgery, immediately after surgery and at the last follow-up. For young patients with low compliance to undergo standing radiographs at immediately post-operation, radiographs taken at 3-month follow-up were used to assess the immediately postoperative correction results. Preoperative computed tomography (CT) scan and three-dimensional reconstruction of the thoracolumbar spine were utilized to record the location, type, and segmentation of hemivertebra. Pedicle size was measured based on preoperative transverse CT scans. The accuracy of pedicle screw placement was assessed using postoperative CT scan. Based on the established grading system, pedicle screw perforations were classified as medial, lateral or anterior and were categorized into four grades: grade 1, ≤ 2 mm; grade 2, 2.1–4.0 mm; grade 3, 4.1–6.0 mm; and grade 4, ≥ 6.1 mm. [20–22] Magnetic resonance imaging (MRI) of the whole spine was also performed to identify the associated intraspinal malformations preoperatively.

The radiographic parameters were measured on the standing erect posteroanterior X-ray planes of the whole spine, including: (1) main curve: the angle between the superior endplate of the vertebra above the hemivertebra and the inferior endplate of the vertebra below the hemivertebra; (2) compensatory curve: measured using the Cobb’s method; (3) coronal balance distance (CBD): the horizontal distance between C7 plumb line and the central sacral vertical line (CSVL); (4) segmental kyphosis (SK): the angle between the superior endplate of the vertebra above the hemivertebra and the inferior endplate of the vertebra below the hemivertebra; (5) thoracic kyphosis (TK): the angle between the superior endplate of T5 and the inferior endplate of T12; (6) lumbar lordosis (LL): the angle between the superior endplate of L1 and the inferior endplate of S1; (7) proximal junctional angle (PJA): the angle between the inferior endplate of the upper instrumented vertebra (UIV) and the superior endplate of the normal vertebra above the hemivertebra [23]; (8) distal junctional angle (DJA): the angle between the superior endplate of the lowest instrumented vertebra (LIV) and the inferior endplate of the vertebra below the hemivertebra [24]; and (9) sagittal vertical axis (SVA): the horizontal distance between the vertical line drawn from the middle of C7 and the superior posterior endplate of the sacrum. All parameters were measured independently by two of the authors, and the average values were applied for further analysis.

In the coronal plane, progression of a newly developed curve above or below the surgical region more than 10° during postoperative follow-up was defined as adding-on deformity [7]. In the sagittal plane, in cases with PJA more than 10° or at least 10° greater than the immediately postoperative value during follow-up, the diagnosis of proximal junctional kyphosis (PJK) was determined [23]. Similarly, distal junctional kyphosis (DJK) was diagnosed as DJA no less than 10°. [24]

Surgical complications were subdivided into implant-related complications and alignment-related complications. Pedicle screw malposition, pedicle fracture and implant prominence were classified into implant-related complications, while proximal adding-on and distal adding-on in the coronal plane and PJK and DJK in the sagittal plane were categorized into alignment-related complications.

### Statistical analysis

All of the parameters were analyzed using standardized statistical software (SPSS version 22.0, Chicago, IL). Continuous data were described as the mean ± standard deviation. Comparisons between the two groups were performed using independent-sample *t* test or Chi-square tests. A statistical difference was set at *P* < 0.05.

## Results

### Demographic data

A total of 57 patients were recruited in this study. There were 24 patients and 33 patients in Group E and Group D, respectively. On average, patients in Group E were 1.9 years younger than Group D (2.3 ± 0.6 years vs. 4.2 ± 0.9 years). No significant difference was found between Group E and Group D in terms of gender, hemivertebra location, preoperative ASA or postoperative follow-up duration (Table 1).

**Table 1:** Demographic and clinical data of the two groups

	Group E (< 3 years)	Group D (3–5 years)	*P* value
Number	24	33	–
Age (years)	2.3 ± 0.6	4.2 ± 0.9	–
Gender (F/M)	13/11	17/16	0.843
Location of hemivertebra			
T10	3 (13%)	4 (12%)	0.954
T11	5 (21%)	6 (18%)	
T12	7 (29%)	9 (27%)	
L1	6 (25%)	7 (21%)	
L2	3 (13%)	7 (21%)	
Preoperative ASA (%)			
1	18 (75%)	27 (82%)	0.533
2	6 (25%)	6 (18%)	
Follow-up duration (m)	75.3 ± 18.1	79.7 ± 19.7	0.393

ASA: American Society of Anesthesiologists

### Comparisons of correction results between Group E and Group D

As shown in Table 2, patients of Group E had shorter operation time and less blood loss during surgery than those of Group D (*P* = 0.003 and *P* = 0.006). The fusion segments were 2.3 ± 0.7 and 3.1 ± 1.2 in Group E and Group D (*P* = 0.005), respectively, indicating that Group E saved 0.8 motion segments on average. Compared with Group D, Group E had smaller pedicles (*P* = 0.045).

**Table 2:** Comparisons of clinical data and radiographic parameters in the two groups

	Group E (*n* = 24)	Group D (*n* = 33)	*P* value
Fusion segments	2.3 ± 0.7	3.1 ± 1.2	0.005
Operation time (min)	185.7 ± 23.3	211.4 ± 35.8	0.003
Blood loss (ml)	198.8 ± 49.5	242.5 ± 61.2	0.006
Pedicle size (mm)	4.4 ± 0.6	4.7 ± 0.5	0.045
Main curve			
Pre-operation (°)	33.5 ± 15.3	41.7 ± 18.5	0.082
Immediately post-op (°)	5.5 ± 2.7	6.9 ± 4.8	0.204
Correction (%)	83.3 ± 21.6	81.2 ± 20.1	0.707
Last follow-up (°)	10.3 ± 3.0	11.5 ± 3.3	0.165
Correction loss (%)	16.3 ± 8.9	8.8 ± 6.4	0.001
Compensatory curve			
Pre-operation (°)	23.5 ± 15.3	27.8 ± 16.4	0.319
Immediately post-op (°)	12.5 ± 6.9	15.3 ± 7.4	0.153
Last follow-up (°)	14.5 ± 7.6	17.7 ± 7.9	0.131
CBD			
Pre-operation (cm)	1.3 ± 1.6	1.5 ± 1.3	0.605
Immediately post-op (cm)	0.2 ± 0.9	0.2 ± 0.8	0.813
Last follow-up (cm)	0.2 ± 1.0	0.1 ± 0.8	0.677
Segmental kyphosis			
Pre-operation (°)	17.4 ± 14.7	24.2 ± 18.5	0.142
Immediately post-op (°)	6.3 ± 3.0	6.9 ± 3.8	0.524
Correction (%)	65.1 ± 23.8	71.7 ± 24.9	0.319
Last follow-up (°)	10.2 ± 3.0	8.2 ± 3.3	0.023
Correction loss (°)	24.5 ± 13.7	5.1 ± 4.1	0.000
TK			
Pre-operation (°)	27.8 ± 15.8	28.5 ± 17.1	0.875
Immediately post-op (°)	25.0 ± 7.7	24.8 ± 8.2	0.926
Last follow-up (°)	26.8 ± 8.4	25.9 ± 8.8	0.699
LL			
Pre-operation (°)	− 38.5 ± 16.3	− 37.2 ± 18.4	0.784
Immediately post-op (°)	− 37.9 ± 17.4	− 38.8 ± 18.8	0.855
Last follow-up (°)	− 39.7 ± 21.2	− 40.3 ± 20.8	0.915
SVA			
Pre-operation (cm)	1.1 ± 1.7	1.0 ± 1.5	0.815
Immediately post-op (cm)	0.7 ± 1.5	0.7 ± 1.6	0.727
Last follow-up (cm)	0.7 ± 1.6	0.6 ± 1.5	0.810

CBD: Coronal balance distance; TK: thoracic kyphosis; LL: lumbar lordosis; SVA: sagittal vertical axis

**Fig. 1:**
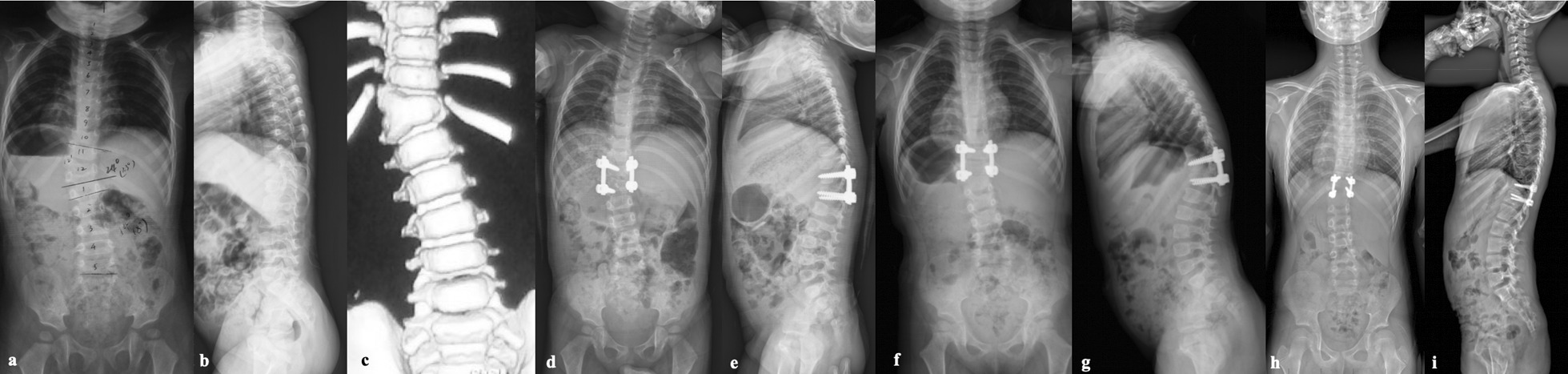
**a–c** A 2-year- and 7-month-old girl with T12 hemivertebra were treated with T12 hemivertebra resection and short segmental fusion. **d**, **e** After surgery, the main curve was corrected from 24° to 5°. **f**, **g** Eleven months later, a new curve including the fusion segments with a Cobb angle of 19° emerged, and distal junctional kyphosis gradually occurred. Bracing treatment was prescribed to this patient. **h**, **i** Six years after surgery, the emerged curve was decreased to 8° by bracing treatment. And distal junctional kyphosis was successfully managed

**Fig. 2:**
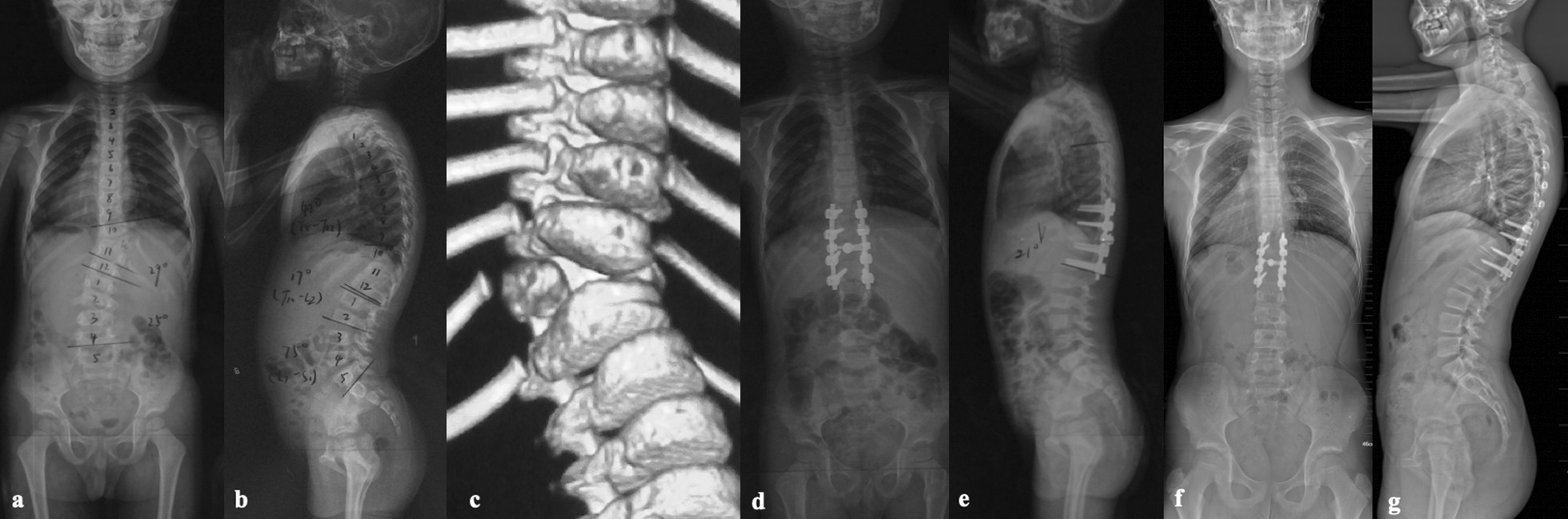
**a–c** A 3-year- and 9-month-old boy with T11 hemivertebra were treated with T11 hemivertebra resection and short segmental fusion. **d**, **e** After surgery, the main curve was corrected from 29° to 3° and the segmental kyphosis was corrected from 17° to 0°. **f**, **g** Eight years after surgery, the trunk showed a well-balanced state in both coronal and sagittal plane

At the time of surgery, patients of Group E had smaller main curve magnitude in the coronal plane than patients of Group D, but the difference was not statistically significant (*P* = 0.082). After surgery, the main curve was corrected from 33.5 ± 15.3° to 5.5 ± 2.7° in Group E and from 41.7 ± 18.5° to 6.9 ± 4.8° in Group D, showing a similar correction rate (83.3% vs. 81.2%, *P* = 0.707) in two groups (Figs. 1 and 2). At the last follow-up, the main curve increased to 10.3 ± 3.0° in Group E and 11.5 ± 3.3° in Group D, demonstrating slightly but significantly more correction loss in Group E (16.3% vs. 8.8%, *P* = 0.001).

Patients of Group E were noticed with less severity of preoperative segmental kyphosis than those of Group D (17.4 ± 14.7° vs. 24.2 ± 18.5°, *P* = 0.142). After surgery, segmental kyphosis was corrected to 6.3 ± 3.0° and 6.9 ± 3.8° in Group E and Group D, respective, indicating a similar correction rate (65.1 ± 23.8% vs. 71.7 ± 24.9%, *P* = 0.319). At the latest follow-up, Group E was observed with more correction loss of segmental kyphosis than Group D (24.5 ± 13.7% vs. 5.1 ± 4.1%, *P* < 0.001).

No significant differences were found between the two groups in terms of compensatory curve, CBD, TK, LL or SVA at any time point (*P* all > 0.05).

### Complications

After surgery, no neurological complication, major vascular complication, pseudoarthrosis or instrumentation breakage was observed in either group. One patient of Group E suffered from superficial infection at 1 week after surgery. After debridement, the wound healed eventually.

According to postoperative CT scans, 9 pedicle screws in 8 patients (33.3%) of Group E and 7 pedicle screws in 5 patients (15.2%) of Group D perforated at least one cortical wall, respectively. Grades of perforations in the two groups are presented in Table 3. Pedicle fracture was observed in 4 patients of Group E and 2 patients of Group D. Additionally, 3 patients of Group E and 1 patient of Group D were noticed with implant prominence. On total, implant-related complications occurred in 15 (62.5%) patients of Group E and 8 (24.2%) patients of Group D, showing a higher incidence of implant-related complications in Group E than that in Group D (*P* = 0.004) (Table 4).

**Table 3:** Grades of perforations in Group E and Group D

	Group E	Group D
Grade 1	6	5
Grade 2	2	2
Grade 3	1	0
Grade 4	0	0

**Table 4:** Complications in Group E and Group D

	Group E (*n* = 24)	Group D (*n* = 33)	*P* value
Implant-related (total)	15 (62.5%)	8 (24.2%)	0.004
Pedicle screw malposition	8	5	
Pedicle cutting-off	4	2	
Implant prominence	3	1	
Superficial infection	1	0	–
Alignment-related			
Proximal and distal adding-on	11 (45.8%)	7 (21.2%)	0.048
PJK and DJK	10 (41.7%)	5 (15.2%)	0.025

“–”: no significant difference; PJK: Proximal junctional kyphosis; DJK: distal junctional kyphosis

Follow-up information regarding the spine profile was assessed on both coronal and sagittal planes. Proximal or distal adding-on deformity was noticed in 11 (45.8%) patients of Group E and 7 (21.2%) of Group D, respectively (*P* = 0.048). In the sagittal plane, PJK or DJK was observed in 10 (41.7%) patients of Group E and 5 (15.2%) patients of Group D, respectively (*P* = 0.025). A higher incidence of alignment-related complication was found in Group E (Table 4). Seven patients with a curvature of adding-on deformity more than 20° and 4 patients with junctional problems received bracing treatment. By the latest follow-up, no revision surgery was required due to alignment-related complications.

## Discussion

To our knowledge, this is the first study to comprehensively compare the correction outcomes and complications between delayed hemivertebra resection (between 3 and 5 years of age) and earlier hemivertebra resection (before 3 years of age). Our results showed that the younger age group (Group E) had similar correction results but greater correction loss and higher complication rate compared with the older age group (Group D).

Hemivertebra resection was accepted as the optimal treatment for CS patients secondary to a hemivertebra [5–7, 9, 15]. With the advancement of pedicle screw fixation technique, excision of hemivertebra could even be performed in very young patients [5, 6, 15]. Ruf et al. [5] reported a 68.9% curve correction in 28 CS patients who were treated by hemivertebra resection at a young age from 15 months to 6 years and 11 months. They found that this procedure was well tolerated even in very young patients and proposed that hemivertebra resection should be performed as early as possible. Chang et al. [25] showed that patients who underwent hemivertebra resection before 6 years old had better correction results than those received surgery after 6 years old. Our study demonstrated an 83.3% and an 81.2% correction rate of main curve in Group E and Group D at immediately post-operation, showing a similar correction rate between the two groups. The equal correction results in the two groups might be attributed to that all patients in our study were treated by hemivertebra excision at a rather young age with similar curve magnitude and flexibility. At the last follow-up, however, more correction loss of main curve and segmental kyphosis was observed in younger age patients (Group E). The main reason might lie in that younger age patients had weaker pedicles which increased the difficulty of maintaining the corrections. Therefore, for patients with mild curve or non-rapid progression curve, postponing hemivertebra resection until 3 years old might be a reasonable alternative choice to obtain long-term sustainable corrections.

Saving motion segments is another major goal particularly for young scoliosis patients who require correction surgery [5, 7, 15]. In young CS patients, the compensatory curve was initially non-structural. Along with the skeletal development, the non-structural compensatory curve might gradually become structural and rigid. In these conditions, more motion segments had to be fused in order to obtain a satisfied correction. Ruf et al. [5] stressed that early hemivertebra resection was important in preserving motion segments. In our study, compared with patients of Group D, an average 0.8 motion segments were saved in younger age patients (Group E). Chang et al. [14] also found that earlier surgery in CS patients could save a mean 1.3 segments. These findings indicated that hemivertebra resection should be performed as early as possible to save motion and growth segments.

Unfortunately, young age was identified as an important factor that might increase the risk of implant-related complications [26]. Ruf et al. [5] reported that 17.9% (5/28) patients experienced this complication after early hemivertebra resection, including 2 pedicle fractures and 3 implant failures. Guo et al. [15] reported 1 pedicle fracture, 1 rod breakage and 2 additional surgeries for pedicle elongation in 39 young CS patients. In our study, implant-related complication occurred in 15 (62.5%) patients of Group E and in 8 (24.2%) patients of Group D, showing a little higher incidence of this unexpected complication than that of the above studies. This difference might be due to our strict definition of implant-related complications in which pedicle screw malposition and implant prominence were included. Additionally, our results further showed that younger age patients (Group E) were more predisposed to experience implant-related complications. The reasons might lie in two factors. First, younger patients commonly had smaller and weaker pedicles (Table 2). Although the safety of pedicle screws instrumentation in pediatric patients has been proven, insertion of pedicle screws remains technically demanding. Perforations might be inevitable particularly in pediatric patients with thin pedicles. Secondly, weaker pedicles, smaller patients’ size and worse soft tissue in younger patients might increase the risk of pedicle fracture and implant prominence.

In addition to implant-related complication, spinal profile in the coronal and sagittal plane is another important issue that has drawn spine surgeon’s attention [6, 16, 27, 28]. Li et al. [6] reported that 10.1% (18/179) patients had postoperative curve progression around the fusion region after thoracolumbar hemivertebra resection and found that greater LIV translation and unsatisfactory LIV horizontalization were responsible for this complication. As for the postoperative sagittal profile, Wang et al. [16] demonstrated an incidence of 18.9% (7/37) of junctional disorders in young CS patients who underwent hemivertebra resection and short fusion. They noticed that misplaced pedicle screws at UIV could lead to postoperative PJK. Our results showed a higher incidence of postoperative spinal malalignment in younger age patients both in the coronal and in the sagittal plane. Since the concentration of strong stress on UIV and LIV following short fusion surgery, pedicle screw malposition at UIV or LIV might increase the risk of alignment-related complications. Notably, application of navigation system in recent years might provide a valuable assistance to decrease pedicle screw malposition. In terms of management of adding-on phenomenon in the coronal plane or junctional problem in the sagittal plane, bracing treatment was conventionally recommended. In our study, adding-on deformities and junctional disorders were all successfully controlled and no revision surgery was required at the latest follow-up.

Most of CS secondary to a hemivertebra may be associated with a more or less pronounced kyphosis [29]. Segmental kyphosis was reported as a risk factor responsible for rapid progression of deformity. In cases with pronounced kyphosis which resulted from a hemivertebra, early surgery is frequently needed to prevent the deformity deterioration. Although our study revealed a higher complication rate in patients before the age of 3 years old, we still highly recommended early hemivertebra resection for patients who were identified with marked segmental kyphosis at any age. For non-kyphotic hemivertebra, delayed surgery might be an appropriate alternative with the goal of avoiding more complications and correction loss.

There were some limitations in our study. Firstly, concerning the young age of the enrolled patients, results about quality of life were not assessed in our study. Secondly, this was a retrospective study and the sample size was relatively small. Thirdly, most patients were skeletal immature at the last follow-up.

In conclusion, both groups had satisfied correction results after hemivertebra resection and pedicle screw instrumentation. Hemivertebra resection resulted in similar correction results in both age groups. However, the rate of complications was higher for the younger age group (before 3 years old) than the older age group (between 3 and 5 years old). Thus, for non-kyphotic hemivertebra, surgery may be delayed till 3 to 5 years of age.

## Data Availability

Please contact author for data requests.

## Abbreviations

CS: Congenital scoliosis
ASA: American Society of Anesthesiologists
SEP: Sensory evoked potential
MEP: Motor evoked potential
CT: Computed tomography
MRI: Magnetic resonance imaging
CBD: Coronal balance distance
CSVL: Central sacral vertical line
SK: Segmental kyphosis
TK: Thoracic kyphosis
LL: Lumbar lordosis
PJA: Proximal junctional angle
UIV: Upper instrumented vertebra
DJA: Distal junctional angle
LIV: Lowest instrumented vertebra
SVA: Sagittal vertical axis
PJK: Proximal junctional kyphosis
DJK: Distal junctional kyphosis
